# Antibiotic Prophylaxis Prescribing Practices for Dental Implant Placement in Croatia: A Questionnaire-Based Cross-Sectional Study

**DOI:** 10.3390/antibiotics14010047

**Published:** 2025-01-08

**Authors:** Mare Ković, Ajka Pribisalić, Joško Viskić, Jure Martinić, Josipa Grubišić, Ante Vardić, Tina Poklepović Peričić

**Affiliations:** 1 Private Dental Office, 21000 Split, Croatia; kovic.mare@gmail.com; 2 Department of Health Studies, University of Split, 21000 Split, Croatia; 3 Department of Public Health, University of Split School of Medicine, 21000 Split, Croatia; 4 Department of Fixed Prosthodontics, School of Dental Medicine, University of Zagreb, 10000 Zagreb, Croatia; viskic@sfzg.hr; 5 Zagreb County Public Health Dental Office, 10000 Zagreb, Croatia; juremartinic98@gmail.com; 6 Study of Dental Medicine, University of Split School of Medicine, 21000 Split, Croatia; josipagrubisic15@gmail.com; 7 Split-Dalmatia County Public Health Dental Office, 21000 Split, Croatia; ante.vardic7@gmail.com; 8 Department of Prosthodontics, Study of Dental Medicine, University of Split School of Medicine, 21000 Split, Croatia; tina.poklepovic.pericic@mefst.hr

**Keywords:** antibiotics, dental implants, Croatia, dentists, cross-sectional, questionnaire

## Abstract

**Background/Objectives**: This study aimed to explore antibiotic prescribing practices for dental implant placement in Croatia. **Methods**: We conducted a cross-sectional questionnaire-based study including dentists in Croatia who perform dental implant therapy. The questionnaire assessed the dentists’ age, working experience, education level, and whether they use antibiotics for dental implant placement, as well as the choice of antibiotics, timing, and reasons for antibiotics use. We used snowball and convenient sampling methods for recruiting dentists. Categorical data were described as absolute numbers and percentages. Differences in the use of antibiotics for specific health conditions were analyzed using Chi-Square, with *p* < 0.05. **Results:** Overall, 74 dentists completed the survey. The dentists used antibiotics either before and after (N = 37, 48.7%), before (N = 21; 27.6%), or after dental implant placement (N = 17, 22.4%). Most used Amoxicillin (N = 47, 61.8%), or Amoxicillin–clavulanic acid (N = 22, 28.9%). Almost all dentists used antibiotics in patients with artificial heart valves (N = 73, 97.3%) and a history of infective endocarditis (N = 74, 98.7%). Also, the dentists reported using antibiotics in patients with artificial joints (N = 52, 69.3%), diabetes (N = 48, 64%), HIV (N = 51, 34.2%), or those on antiresorptive drugs (N = 46, 61.3%), with 17 dentists (22.7%) prescribing antibiotics to all (*p* < 0.001). The main reasons for antibiotic prophylaxis were preventing complications at the implant site (N = 56; 73.7%) and reducing the early implant failure rate (N = 32; 42.1%). Around one-third of the dentists (34.2%) used antibiotics for their own safety. **Conclusions**: Croatian dentists may be overprescribing antibiotics during dental implant placement. Clear recommendations concerning antibiotic prophylaxis for dental implant therapy are needed to make well-informed clinical decisions.

## 1. Introduction

Antibiotic prophylaxis is the preventative administration of antibiotics during and after surgical procedures to reduce the risk of bacteriemia and serious complications, particularly bacterial infection [1,2].

In dental medicine, antibiotic prophylaxis is indicated before dental procedures involving the manipulation of the oral mucosa, gingival area, or periapical tissue, including tooth extractions, biopsies, and dental implant placement [1,2,3].

For surgical procedures, antibiotic prophylaxis has been recommended in patients with specific health issues like those with impaired immune systems; for patients with a high risk of infective endocarditis, artificial joints; or those having received radiotherapy of the head and neck, as well as those on bisphosphonates or antiangiogenic drugs. Despite limitations in the available guidance on the use of antibiotics in dentistry, recommendations do not support the routine administration of prophylactic antibiotics in healthy individuals. Yet, antibiotics are given if a surgical procedure is performed in an infected site; for lengthy, extensive surgical procedures; or the implantation of foreign materials regardless of the patient’s health status [1,3,4,5].

The rationale for antibiotic prophylaxis in dental implant therapy, however, stems from the understanding that local bacterial infections can significantly compromise the success of dental implant therapy, leading to implant failure and other complications, such as implant rejection, peri-implantitis, and surgical site infection [1,2]. That is why various antibiotic regimens have been prescribed for dental implant placement worldwide. However, the necessity and appropriateness of routine antibiotic use for healthy individuals in dental implant surgery have been subjects of ongoing debate.

According to the American Heart Association (AHA) [3], the recommended antibiotic for prophylaxis in dental medicine, in general, is Amoxicillin, while in the case of allergy, Azithromycin or Clarithromycin should be prescribed [3,5].

The American Dental Association (ADA) recommends following the AHA 2021 guidelines [3,5]. The AHA and other professional organizations, like the Dutch Orthopaedic and Dental Societies or the Academy of American Orthopedic Surgeons, along with the available evidence synthesis and evidence concerning everyday practice, recommend a more conservative approach for healthy patients undergoing routine dental surgical procedures, including implant placement. All suggest against routine antibiotic prophylaxis unless specific risk factors and certain medical conditions are present, predisposing patients to infections [3,5,6,7,8,9].

Despite the positive aspects that prophylaxis may bring, the rate of bacterial antibiotic resistance is increasing. It represents a major concern worldwide because of the significant risks of antibiotic-resistant bacteria and adverse drug reactions [1,2,10]. The World Health Organization (WHO) has emphasized the importance of responsible antibiotic use to combat the global issue of antibiotic resistance. The Spanish Society of Implants has also developed clinical practice guidelines emphasizing responsible antibiotic prescribing based on the current scientific evidence to mitigate the risk of antimicrobial resistance [11,12]. Dental practitioners are encouraged to evaluate the necessity of antibiotic prophylaxis on a case-by-case basis, considering patient-specific factors and the type of surgical procedure being performed [1,2,11].

Research has shown mixed results regarding the efficacy of prophylactic antibiotics in preventing infections following dental implant surgery. Two systematic reviews indicated that a single preoperative dose of antibiotics might reduce the risk of early implant failure, particularly in patients with higher risk profiles [9,13]. A study involving diabetic patients demonstrated that preoperative antibiotic prophylaxis significantly increased the success rate of dental implant therapy with this condition [10]. However, despite the evidence supporting the use of prophylactic antibiotics to prevent local infections, the scale of the benefits appears limited. The widespread use of antiseptics and antibiotics during dental implant placement, despite the possible reduction in the incidence of local infection, does not necessarily prevent implant failure, suggesting that routine prophylaxis is not clinically justified in healthy patients and should only be considered for specific patient groups [14,15]. An earlier systematic review highlighted that a single preoperative dose of antibiotics might reduce the risk of early implant failure in healthy patients, yet the overall impact remains inconclusive [9]. Other studies, however, suggested that the benefits of antibiotic prophylaxis are marginal and that the routine use of antibiotics may contribute to the development of antibiotic resistance [14,16]. A multicenter observational study found that while antibiotics are frequently prescribed, the evidence supporting their use is insufficient to make definitive recommendations [14].

The heterogeneity of the available evidence on antibiotic prophylaxis during implant surgery contributes to difficulties in formulating clear and generally acceptable guidelines [14]. The observed inconsistencies in practices across different regions call for standardized protocols on antibiotic use in dental implant surgery [17].

The current landscape on antibiotic prescribing patterns in dental implant surgery varies significantly [18,19], with unclear and contradictory findings from the available evidence base leading to unnecessary antibiotic prescriptions [6]. This calls for urgent consensus on the use of antibiotics in dental implantology that can be universally applied, ensuring that decisions are based on the best available scientific evidence and tailored to individual patient needs.

Therefore, this study aimed to explore Croatian dentists’ attitudes and practices regarding the use of antibiotics during dental implant placement. More specifically, we aimed to investigate whether dentists prescribed antibiotics for implant therapy and, if so, when (before, after, or before and after dental implant placement). Also, this study examined the type of antibiotics dentists usually use, the reasons for their administration, potential correlations between antibiotic prescribing practices and patients’ health conditions, and specific dentists’ characteristics.

## 2. Results

A total of 84 dentists responded to the questionnaire, resulting in a response rate of 28%. After excluding eight dentists because dental implant therapy was not part of their usual practice, the final sample for the analysis consisted of 76 dentists. Most were general dentists (68.4%), and most worked in private practice (86.8%). Of all the dentists with specialization (31.6%), half were oral surgeons. The majority of the participating dentists were aged 31 to 50 years (61.8%), with either 11 to 20 years of experience (39.5%) or more than 21 years of clinical experience (28.9%). Only three dentists were 61 or older (Table 1).

Regarding the use of antibiotics for dental implant placement, almost half of all the participating dentists (48.7%) reported prescribing antibiotics both before and after implant placement. The others prescribed antibiotics before (27.6%) or after (22.4%) dental implant placement. Only one dentist (1.3%) reported not to be prescribing antibiotics for dental implant placement (*p* < 0.001, compared to those who prescribe). As for the type of antibiotic dentists use, Amoxicillin was the most commonly prescribed (61.8%), followed by Amoxicillin–clavulanic acid (28.9%). Some dentists, however, reported using Clindamycin and Erythromycin (1.3%). One dentist reported using either Amoxicillin–clavulanic acid or Clindamycin (Table 2).

**Figure 1:**
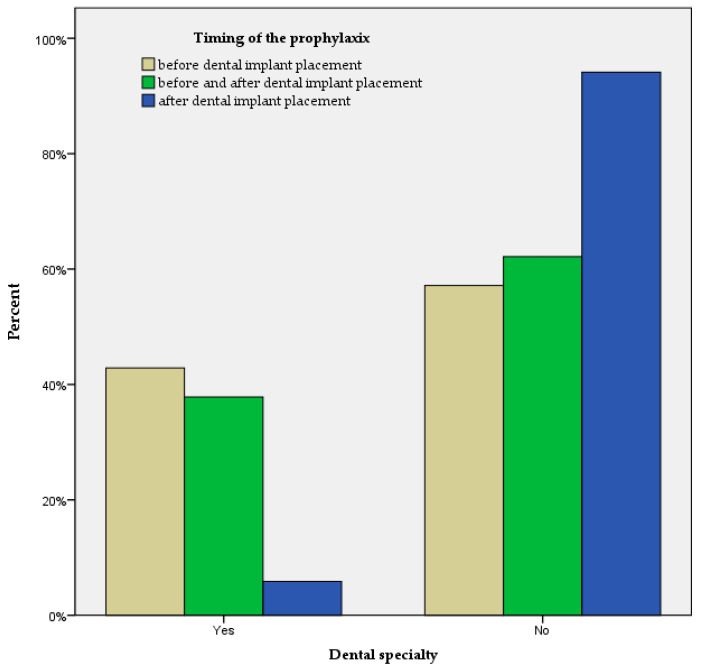
Timing of the prophylaxis with regard to dental specialty (N = 75).

The differences between the demographic characteristics and the timing and type of antibiotic prophylaxis were analyzed. Only one significant difference was found; between the timing of the prophylaxis and overall dental specialty (*p* = 0.031, Fisher exact test) (Figure 1).

Pairwise post hoc *p*-values were significant for the differences between the “Before and After” vs. “After” timing of prophylaxis and dental specialty (*p* = 0.013), and differences between the “Before” vs. “After” timing of prophylaxis and dental specialty (*p* = 0.011). Differences between the “Before” vs. “Before and After” timing of prophylaxis and dental specialty were not significant (*p* = 0.460).

Despite some dentists considering antibiotic prophylaxis should be given to all patients, regardless of their health state, the majority (77.3%) do not consider antibiotic prophylaxis necessary for all patients (*p* < 0.001). Considering specific indications for antibiotic prophylaxis during dental implant placement, there was a high level of agreement among the dentists for the use of antibiotics in high-risk patients, such as patients with artificial heart valves and patients with a history of infective endocarditis, with 97.3% and 98.7% dentists considering prophylaxis a necessity, respectively (*p* < 0.001). Also, a significant number of dentists (77.3%) would recommend antibiotic prophylaxis for patients with transplanted organs compared to those who would not (*p* < 0.001). Conditions like HIV, dialysis, built-in pacemaker heart devices, artificial joints, and diabetes mellitus were also considered indications for antibiotic prophylaxis (64.0–69.3%, all *p* < 0.010). Similar approaches were observed for patients on antiresorptive and antiangiogenic therapy (61.3%, *p* = 0.027) (Table 3).

The main reason for antibiotic prophylaxis in dental implant placement was to prevent the frequency and intensity of complications at the implant site, specifically infection (N = 56; 73.7%). The dentists also considered antibiotic prophylaxis to reduce the rate of early implant failure (N = 32; 42.1%). Some, however, reported using antibiotic prophylaxis in dental implant therapy for their sense of security (N = 26; 34.2%). No other reasons for antibiotic prophylaxis were provided.

According to the dentists’ responses, their source of information regarding antibiotic prophylaxis in dental implant therapy was the most recently published guidelines (N = 29; 38.2%). Also, the dentists reported learning about antibiotic prophylaxis during continuing education courses (N = 26; 34.2%), while a smaller number of the dentists received information on the use of antibiotics for dental implant therapy during their formal college education (N = 19; 25%). One dentist reported having learned about the topic during master’s studies (1.3%), and one dentist (1.3%) reported to have been learning about antibiotics in dental implant therapy by combining all the sources mentioned above (*p* < 0.001).

## 3. Discussion

This study found that almost half of all the dentists prescribe antibiotics before and after implant placement, with around a quarter of dentists prescribing antibiotics before or after dental implant placement. These findings contradict the current guidelines, which recommend against routine antibiotic prophylaxis for healthy patients undergoing dental implant surgery [3,5,7]. However, our study’s results align with similar studies worldwide, showing significantly variable prescribing practices, often contradictory to the available recommendations. In Sweden, most dentists routinely prescribe prophylactic antibiotics before implant surgery [20]. Comparably, dentists from the UK prescribe antibiotics one hour preoperatively [21], while in Italy, a high rate of antibiotic prophylaxis during implant placement has been observed [2,22].

Furthermore, contrary to the available guidance, around one-quarter of the dentists from our study prescribe antibiotics even to completely healthy patients. Sanchez et al. found similar results, where almost two-thirds of the study participants did not follow the current prophylactic regimen and almost always recommended antibiotic prophylaxis [22]. In the UK, more than 70% of the surveyed dentists who work with dental implants routinely prescribe prophylactic antibiotics to all patients [21].

The results suggest that the presence of specific medical conditions significantly influences the decision to prescribe antibiotic prophylaxis among the surveyed dentists, with almost all the dentists having prescribed prophylaxis to patients with artificial heart valves and a history of infective endocarditis. This is in line with the available AHA guidelines that support using antibiotics before invasive dental procedures [3] for medical conditions like impaired immune system, a history of infective endocarditis, and conditions associated with a risk of developing infective endocarditis [3,5].

Likewise, most dentists prescribe antibiotic prophylaxis for patients with artificial joints. Recommendations concerning antibiotic prophylaxis for invasive dental treatment in patients with artificial joints have undergone considerable changes. The guideline developers often considered time from surgical procedures, whereas two years from surgery were considered a threshold for a long time. The recent guidelines considered a balance between the benefits and harms of antibiotic prophylaxis [5] and now recommend against antibiotic prophylaxis in patients with artificial joints, regardless of their immune status [6,23].

Also, we found that the dentists would prescribe antibiotic prophylaxis to patients with built-in pacemakers, although relevant authorities, like the AHA and the American College of Cardiology, consider this unnecessary. The recommendations for dental procedures are now strongly against antibiotic prophylaxis for dental procedures in these patients [3,5,24].

The dentists from our study would recommend antibiotic prophylaxis for patients with transplanted organs. However, it is considered that prophylaxis should not be given routinely, only based on the patient’s medical history. In patients with transplanted organs, dentists are encouraged to consult with the patient’s physician to assess the patient’s health status and determine if antibiotic prophylaxis is needed [25]. More importantly, in the pre-transplantation period, dentists should ensure that all the potential risk factors that could consequently lead to infection in the post-transplantation period are treated or removed from the mouth. Emphasis is given on the influence of oral infection on the prognosis of a transplanted organ [5,26].

More than half of the dentists in our study believe that antibiotic prophylaxis is necessary for patients with diabetes mellitus. However, there are no available guidelines on the use of antibiotics for invasive dental procedures in patients with diabetes [5]. There is evidence suggesting that to improve implant survival and reduce postoperative complications, supportive therapy, such as prophylactic antibiotics and chlorhexidine mouth rinse, should be used [10,27]. However, there is no strong evidence of the need for antibiotics in patients with diabetes. Dental implant placement is considered safe for patients with well-controlled diabetes and even for those with moderately controlled diabetes. There are also no recommendations for antibiotic use in patients with poorly controlled diabetes; however, since the prognosis of dental implant therapy in these patients is unpredictable, with delayed osseointegration and a higher risk of dental implant failure, antibiotics may be given in these patients postoperatively [5].

HIV was also one of the reasons for antibiotic prophylaxis among our dentists. While HIV is an immuno-suppressive disease, the current ADA recommendation is to get in contact with the patient’s physician to discuss the appropriateness of antibiotic prophylaxis based on the patient’s health state. Otherwise, antibiotics may predispose patients to adverse drug effects and superinfections and should not be used by default [5].

When asked about the patients on antiresorptive therapy and those on antiangiogenic therapy, dentists in our study prescribe antibiotic prophylaxis for both patient groups. This is contrary to the guidelines about managing dental patients on these specific therapeutical modalities, where not only are antibiotics not recommended, but they are proven to interact with vitamin K antagonists and change patients’ INR levels [28,29].

Similarly to our results, a study conducted in Jordan revealed that while some dentists adhere to established guidelines, others prescribe antibiotics based on personal experience rather than evidence [18]. A similar trend was observed in Saudi Arabia, with a notable percentage of dentists believing that antibiotics are necessary for implant surgery in general despite limited supporting evidence [19].

Amoxicillin was the most frequently used antibiotic in our study. The available guidelines recommend Amoxicillin as the first choice for prophylactic purposes before dental procedures in at-risk patients [5,30].

Besides Amoxicillin, our participants also prescribed Amoxicillin with clavulanic acid, Clindamycin, and Erythromycin. This is similar to the UK study, where almost half of dentists routinely prescribed 3 g Amoxicillin. Other antibiotics used in the UK study were Clindamycin, Amoxicillin with clavulanic acid, Metronidazole, and Primcillin [21].

Some dentists reported using Clindamycin, although their reasons for using Clindamycin, whether as a first-choice antibiotic or an alternative due to penicillin allergy, remain unclear. Nevertheless, this indicates a lack of awareness and use of the current guidelines since the recommendations from 2021. advise against the prophylactic use of Clindamycin [3,5] because of its high likelihood of severe adverse events that strongly outweigh the possible benefits of this antibiotic [3].

In cases of reported allergy to Amoxicillin, the guidelines recommend using Azithromycin or Clarithromycin. Surprisingly, none of the dentists in our study mentioned any of these. Although the questionnaire did not necessarily offer all the available antibiotics, this specific question allowed for multiple answers and an opportunity to add other types of antibiotics if some were not mentioned. This could be regarded as a limitation of our study. However, it may also indicate the actual situation because none of the dentists used the option to add an antibiotic, neither azithromycin nor Erythromycin. Considering the serious adverse events that some antibiotics may cause, the severity of the patient’s medical conditions, and the possible influence oral infections may have on patients’ overall health, there is a significant responsibility on dentists, one that they should be made aware of. Relying on good practice guidelines should be the key approach to cope with these challenges sufficiently. Therefore, it is essential to develop and disseminate clear recommendations on antibiotics use for specific medical conditions and make them easily accessible to dentists.

The main reasons for the dentists in our study to prescribe antibiotic prophylaxis were to prevent complications at the implant site and to reduce the rate of early implant failure. Some even use antibiotics for their sense of security. The reported reasons are intriguing, given that antibiotics are usually given to patients with systemic conditions, while the observed reasons for their use seem local. The defensive approach concerning the use of antibiotics may be due to conflicting study results, with some studies suggesting benefits from antibiotic prophylaxis [9,13]. In contrast, others claim evidence is not clinically meaningful [14,15,31].

The dentists in this study are mostly informed about antibiotic prophylaxis in dental implant therapy from the recent guidelines and continuing education courses, while a smaller proportion relied on formal college education. A study conducted in the UK showed that almost half of their dentists learned about prophylaxis from their postgraduate training courses [21]. Similar results come from a study by Al-Kattan et al. and a systematic review by Bernabeu-Mira, both showing that the primary source of dentists’ information is postgraduate courses [9,19]. They show the dentists’ interest in continuing education and their willingness to be informed about the latest evidence and most recent evidence-based recommendations for everyday practice. These findings are crucial to underline the need to develop new guidelines and identify the timing for education and the target population.

The main limitations of this study include sample size and its representativeness. The sample size may not be optimal, and the sampling method may lack probability because it may have included only dentists willing to complete an online questionnaire, biasing towards younger colleagues or those more prone to this topic. Considering the study design and the method of collecting data through a questionnaire, objectivity may be questionable, and the findings of this study should not be regarded as solid evidence.

Nevertheless, despite the limitations, this study’s findings provide valuable local data concerning antibiotic prescribing practices, which has not, to the best of our knowledge, been conducted in Croatia so far. These may be a helpful basis for defining implications for future studies and developing clinical practice guidelines on the use of antibiotics in dental implant therapy. Also, this study gives valuable insight into local needs regarding additional education, raising awareness about rational antibiotic use, and the availability of good quality clinical practice guidelines.

Future studies could mitigate this study’s limitations by using larger and more representative samples. Also, it would be worthwhile to understand factors that shape dentists’ views and practices in more depth through a qualitative study. Evaluating electronic patient health records, including antibiotic prescribing frequencies, rates of dental implant complications, and failure, would clarify local practices better and investigate the potential association between antibiotic prophylaxis and the frequency of complications and dental implant failure rates.

In practice, the results of this study show a divergence in the dentists’ antibiotic prescribing, which is a big concern considering all possible challenges associated with antibiotic overuse. Hence, educational institutions, national bodies, and other professional associations may use this study’s findings to raise awareness of antibiotics and the use of clinical practice guidelines on antibiotics in dental implant therapy.

## 4. Materials and Methods

### 4.1. Study Design

This cross-sectional questionnaire-based study was conducted from May to September 2024. The questionnaire was designed specifically for this study and compiled based on the available research [14,18,19].

The online questionnaire was constructed using Google Forms and subsequently tested to ensure clarity and functionality. We pilot-tested the questionnaire to assess the questionnaire’s suitability by inviting five dentists from the Study of Dental Medicine in Split, Croatia, to complete the questionnaire and provide comments. The results from questionnaire testing were not included in the analyses. The suggestions included changes to the content of the questions, which we adopted.

The questionnaire was designed for Croatian dentists, specialists, and general practitioners routinely engaged in dental implant therapy. Therefore, only dentists who performed dental implant therapy as a part of their usual clinical practice were considered eligible for participation.

A hybrid approach combining convenience and snowball sampling was employed to gather responses. Initially, the link to the survey questionnaire was distributed via email to dentists whose contact information was obtained through personal contacts and professional networks. Subsequently, these dentists were encouraged to forward the link to their colleagues specializing in dental implant therapy, expanding the survey’s reach.

Information about the size of the target population was derived from the Croatian Dental Chamber (available at: https://www.hkdm.hr, accessed on 16 April 2024) data on the official number of registered dentists in Croatia. Based on the assumption that the population size is approximately 4500, the desired precision of the estimate being 0.05, and the confidence level of 0.95, we calculated the minimal sample size using an online calculator (https://epitools.ausvet.com.au, accessed on 16 April 2024). The calculated sample size included 234 dentists. Although we expected not all dentists to work with implants, we still sent the questionnaire to 300 contacts.

Participation in this study was voluntary and anonymous. In the online questionnaire, before beginning the survey, all the participants were provided with a written explanation detailing the study’s purpose and the measures to preserve their anonymity. The participants were also informed that by completing the questionnaire, they provided informed consent to participate in the study. After that, they were allowed to open and complete the questionnaire.

The study was approved by the Ethics Committee of the University of Split School of Medicine in Croatia on 19 May 2024 (Class: 029-01/24-02/0001; Nr.: 2181-198-03-04-24-0093) and was conducted in full accordance with the General Data Protection Regulation, which ensures that all the collected data, as well as the identities of the respondents, are kept completely anonymous. The study report followed the Strengthening the Reporting of Observational Studies in Epidemiology (STROBE) guidelines [32].

### 4.2. Questionnaire

The questionnaire comprised two sub-sections and a total of seventeen questions. The first sub-section consisted of general socio-demographic and professional questions, including gender, age, years of clinical experience, level of education, workplace, and specialist training (Q1–Q7). The second sub-section included questions that assessed the participants’ clinical views on the use, type, and reasons for antibiotics during dental implants’ surgical placement procedure (Q8–Q17). Most of the questionnaire consisted of multiple-choice questions with the possibility of choosing only one answer. Two questions left the possibility of multiple answers (Q13 and Q14), while four questions were open-ended and asked the respondents to write the answers themselves (Q3, Q6, Q10, and Q17). The completion of the questionnaire required approximately five minutes with all the answers mandatory. The full questionnaire is available in the Supplementary Materials.

### 4.3. Data Analysis

The dentists’ responses were collected in an Excel spreadsheet (Ver. Office 2007, Microsoft, Redmond, Washington, DC, USA), subsequently coded, and analyzed using SPSS version 26 (SPSS, IBM Corp, Armonk, NY, USA). All the categorical variables were described using absolute numbers and percentages. A chi-square test was used to analyze categorical data, namely the goodness-of-fit test for testing the distribution of a single variable. The observed frequencies of a single categorical variable were compared to the theoretical frequencies (e.g., in the case of two categories, the null hypothesis for this test would be that the observed distribution of the variable is not significantly different from the expected 50/50 distribution). Fisher exact test was used for testing the association between the two categorical variables due to expected cell counts less than 5, and also for the calculation of pairwise post hoc *p*-values. The significance level was set at *p* < 0.05 (two-sided).

## 5. Conclusions

The responses obtained from this sample suggest that dentists in Croatia may be overprescribing antibiotics in dental implant therapy, including the use of antibiotics before and after dental implant placement, and antibiotics prescribing in healthy patients, as well as in those with health conditions not requiring antibiotic prophylaxis.

Clear recommendations on the use of antibiotics for dental implant therapy, particularly in patients with specific health conditions, are needed to make well-informed clinical decisions.

## Figures and Tables

**Table 1:** Dentists’ demographic characteristics (N = 76).

	N (%)	*p*-Value
Gender		0.359
Male	34 (44.7)	
Female	42 (55.3)	
Age (in years)		<0.001
25–30	10 (13.2)	
31–40	25 (32.9)	
41–50	22 (28.9)	
51–60	16 (21.1)	
61+	3 (3.9)	
Work Experience (in years)		0.007
0–5	11 (14.4)	
6–10	13 (17.1)	
11–20	30 (39.5)	
21+	22 (28.9)	
Education Level		<0.001
DMD	51 (67.1)	
PhD	13 (17.1)	
Master’s Degree	12 (15.8)	
Workplace		<0.001
Public Health Practice	9 (11.8) 67 (88.15)	
Private Practice		
Dental Specialty		0.001
Yes	24 (31.6)	
No	52 (68.4)	
Specialty Field		<0.001
None	52 (68.4)	
Oral Surgery Prosthodontics Periodonotology	12 (15.8) 7 (9.2) 5 (6.6)	

DMD–Dental Medicine Doctor; PhD–doctorate degree; level of significance *p* < 0.05.

**Table 2:** Timing and type of antibiotic prophylaxis used for dental implant placement, N = 76.

	N (%)	*p*-Value
Timing of the prophylaxis		<0.001
Before	21 (27.6)	
After	17 (22.4)	
Before and after	37 (48.7)	
Never	1 (1.3)	
Type of antibiotics		<0.001
Amoxicillin	47 (61.8)	
Amoxicillin and Clavulanic acid	22 (28.9)	
Clindamycin	4 (5.3)	
Erythromycin	1 (1.3)	
Amoxicillin and Clavulanic acid or Clindamycin	1 (1.3)	
Does not use antibiotics	1 (1.3)	

Level of significance *p* < 0.05.

**Table 3:** Antibiotic prophylaxis practices for dental implant placement in relation to patients’ health condition (N = 75).

Patient Condition	Yes N (%)	No N (%)	χ^2^	*p*-Value
All patients	17 (22.7)	58 (77.3)	22.41	<0.001
Artificial heart valves	73 (97.3)	2 (2.6)	67.21	<0.001
History of infective endocarditis	74 (98.7)	1 (1.3)	71.05	<0.001
Transplanted organs	58 (77.3)	17 (22.7)	22.41	<0.001
Built-in pacemaker heart device	50 (66.7)	25 (33.3)	8.33	0.003
HIV	51 (68.0)	24 (32.0)	9.72	0.001
Dialysis	50 (66.7)	25 (33.3)	8.33	0.003
Artificial joints	52 (69.3)	23 (30.7)	11.21	0.001
Antiangiogenic therapy	46 (61.3)	29 (38.7)	3.85	0.027
Diabetes mellitus	48 (64.0)	27 (36.0)	5.88	0.010
Antiresorptive therapy	46 (61.3)	29 (38.7)	3.85	0.027

χ^2^ chi square test; level of significance *p* < 0.05.

## Data Availability

Raw data generated by extractions from the questionnaire and all the relevant data included in our analyses are available upon request from the corresponding author.

## Supplementary Materials

The following supporting information can be downloaded at https://www.mdpi.com/article/10.3390/antibiotics14010047/s1. The questionnaire.
