# Supplementary material for: Antibiotic Prophylaxis Prescribing Practices for Dental Implant Placement in Croatia: A Questionnaire-Based Cross-Sectional Study
Source: Antibiotics (Basel). 2025 Jan 8;14(1):47. doi: 10.3390/antibiotics14010047 (PMC11763041; doi:10.3390/antibiotics14010047)
Supplement: Supplementary file 1 [file antibiotics-14-00047-s001.zip › antibiotics-3354393-supplementary.pdf]

Supplementary file; The questionnaire

General socio-demographic questions		
1.	Gender	- M<br>- F
2.	Age<br>(Please, chose one of the answers)	- 25-30<br>- 31-40<br>- 41-50<br>- 51-60<br>- 61+
3.	Years of clinical experience in dentistry<br>(Please write a number)	
4.	Education<br>(Please, chose one of the answers)	- Dental Medicine Doctor<br>- Master's Degree<br>- PhD
5.	Did you finish specialization?	- YES<br>- NO
6.	If you have answered „YES“ to the previous question, please write which dental specialty training did you finish.	
7.	Workplace (Please, chose one of the answers)	- Free healthcare (state subsidized)<br>- State subsidized<br>- Private office<br>- Hospital<br>- Other
Questions concerning antibiotic prophylactic for dental implant placement		
8.	Do you perform implant dentistry routinely in everyday clinical practice?	- YES<br>- NO
9.	If you answered „YES“ to previous question, do you prescribe antibiotics for dental implant placement procedures?	- YES<br>- NO<br>- Sometimes<br>- Other (Please, explain)
10.	If you have answered „Sometimes“ to the previous question, please elaborate.	
11.	When do you prescribe antibiotics during the dental implant placement procedures?<br>(Please, chose one of the answers)	- Before placement<br>- After placement<br>- Before and after placement<br>- I don't prescribe antibiotics for implant placement

12.	Where do you get information about antibiotic prophylaxis from?	- Undergraduate education<br>- Postgraduate education<br>- Current clinical practice guidelines<br>- Continuing medical education courses<br>- Expert societies newsletter<br>- Other (Please, explain)
13.	For which of the following medical conditions would you prescribe antibiotic prophylaxis during dental implant placement procedure (You may choose more than one answer)?	- Infective endocarditis<br>- Artificial heart valves<br>- Pacemaker<br>- Artificial joints<br>- Diabetes mellitus<br>- Transplanted organs<br>- Patients on antiresorptive therapy<br>- Patients on antiangiogenic therapy<br>- HIV<br>- Dialysis<br>- I never prescribe prophylaxis<br>- Other (Please, explain)
14.	Please state which antibiotics do you prescribe for prophylaxis during dental implant placement.<br>(You may choose more than one answer)	- Amoxicillin<br>- Ampicillin<br>- Metronidazole<br>- Clindamycin<br>- Cephalexin<br>- Erythromycin<br>- Amoxicillin + Clavulanic Acid<br>- I don't prescribe antibiotic prophylaxis<br>- Other
15.	If you have chosen the answer „Other“ to the previous question, please elaborate.	
16.	In general, why do you prescribe antibiotic prophylaxis for dental implant placement?	- To reduce the risk of implant failure<br>- To reduce the infections at the implant site<br>- To reduce the frequency and intensity of complications at the implant site<br>- To feel safe<br>- Other
17.	If you have answered „Other“ to the previous question, please elaborate your reasons for prophylaxis during dental implant placement.	
